# Neonatal vitamin A supplementation associated with a cluster of deaths and poor early growth in a randomised trial among low-birth-weight boys of vitamin A versus oral polio vaccine at birth

**DOI:** 10.1186/1471-2431-14-214

**Published:** 2014-08-28

**Authors:** Najaaraq Lund, Sofie Biering-Sørensen, Andreas Andersen, Ivan Monteiro, Luis Camala, Mathias Jul Jørgensen, Peter Aaby, Christine Stabell Benn

**Affiliations:** 1 Research Center for Vitamins and Vaccines (CVIVA), Bandim Health Project, Statens Serum Institut, Copenhagen, Denmark; 2 Department of Infectious Diseases, Aarhus University Hospital, Aarhus, Denmark; 3 Bandim Health Project, Indepth Network, Bissau, Guinea-Bissau; 4 Maternidade, Hospital Nacional Simão Mendes, Bissau, Guinea-Bissau; 5 OPEN, Institute of Clinical Research, University of Southern Denmark/Odense University Hospital, Odense, Denmark

**Keywords:** Vitamin A supplementation, Oral polio vaccine, Neonate, Cluster, Mortality, Growth

## Abstract

**Background:**

The effect of oral polio vaccine administered already at birth (OPV0) on child survival was not examined before being recommended in 1985. Observational data suggested that OPV0 was harmful for boys, and trials have shown that neonatal vitamin A supplementation (NVAS) at birth may be beneficial for boys. We set out to test this research question in a randomised trial.

**Methods:**

The trial was carried out at the Bandim Health Project, Guinea-Bissau. We planned to enrol 900 low-birth weight (LBW) boys in a randomised trial to investigate whether NVAS instead of OPV0 could lower infant mortality for LBW boys. At birth, the children were randomised to OPV (usual treatment) or VAS (intervention treatment) and followed for 6 months for growth and 12 months for survival. Hazard Ratios (HR) for mortality were calculated using Cox regression. We compared the individual anthropometry measurements to the 2006 WHO growth reference. We compared differences in z-scores by linear regression. Relative risks (RR) of being stunted or underweight were calculated in Poisson regression models with robust standard errors.

**Results:**

In the rainy season we detected a cluster of deaths in the VAS group and the trial was halted immediately with 232 boys enrolled. The VAS group had significantly higher mortality than the OPV0 group in the rainy season (HR: 9.91 (1.23 – 80)). All deaths had had contact with the neonatal nursery; of seven VAS boys enrolled during one week in September, six died within two months of age, whereas only one died among the six boys receiving OPV (p = 0.05). Growth (weight and arm-circumference) in the VAS group was significantly worse until age 3 months.

**Conclusion:**

VAS at birth instead of OPV was not beneficial for the LBW boys in this study. With the premature closure of the trial it was not possible to answer the research question. However, the results of this study call for extra caution when testing the effect of NVAS in the future.

**Trial registration:**

http://www.clinicaltrials.gov NCT00625482. Registered 18 February 2008.

## Background

In low income countries a policy of providing neonatal vitamin A supplementation (VAS) is currently under debate. Four randomised trials from Africa and one from Nepal have shown no overall effect on mortality of neonatal VAS [1-5]. Three trials from South East Asia have reported a beneficial effect [6-8]. Several of the trials suggested that while VAS conferred few benefits or even a negative effect for girls, it had a positive effect in boys [1,2,6,8]. From 1985 WHO recommended a dose of oral polio vaccine at birth (OPV0) in addition to the three doses at 6, 10 and 14 weeks of age (OPV1-3). This policy was introduced to improve coverage and immune responses [9-13]. The effect of OPV at birth on overall child mortality was never studied.

The Bandim Health Project (BHP) has worked in Guinea Bissau since 1978 and has examined non-specific and sex-differential effects on mortality of childhood interventions. From 2002–2004 when BHP was conducting a trial of neonatal VAS to normal birth weight children, OPV was lacking for several periods [14] and some of the enrolled children did not get the recommended OPV0. Surprisingly, boys who did not receive OPV0 only had a third of the mortality of boys who got the vaccine. The tendency was slightly opposite in girls, resulting in a highly significant interaction between OPV at birth and sex (p = 0.006). We also studied the effect of OPV0 on the immune response to BCG vaccine; both sexes had a dampened immune response to BCG if they received OPV together with BCG [15].

Based on these results we hypothesised that newborn LBW boys might benefit from receiving VAS at birth instead of OPV0, and we conducted a randomised trial to test that hypothesis. As the previous studies suggested a harmful effect of VAS in girls [2], only boys were randomised to receive VAS or OPV0. Girls were enrolled in another trial. The trial proceeded as planned from February 2008 until November 2008 when the study supervisor noted a bulk of death reports. Seven boys born between 28 August and 16 September 2008 had died before the 2 months visit. Among the seven deaths six had received VAS. This looked like a cluster and the PI decided to halt the trial to examine possible causes and avoid continuing an intervention which potentially had negative effects.

## Methods

### Setting

The BHP runs a Health and Demographic Surveillance System (HDSS) in six districts of Bissau, the capital of Guinea-Bissau. Since 2002 the BHP has followed a cohort of LBW children from the whole capital. All newborn children weighing less than 2.5 kg at discharge from the maternity ward of the national hospital (NH) are invited to participate. At the time of the trial, 13% of the children born at the NH were LBW. The children and their mothers are driven home from the hospital. A map is drawn describing the localisation of their houses, GPS coordinates are recorded, and a photo of the house and the mother is taken to ensure that the team will be able to localise the child at subsequent visits. When a child moves, a relative or a neighbour takes the team to the new address. In this way very few children are lost to follow up. LBW children living inside the BHP study area who are born at home are recruited when they come for their first vaccinations at one of the three health centres in the study area. In Guinea-Bissau LBW children do not receive BCG at birth, but are told to come back when they have gained weight, and they typically get BCG together with the DTP and OPV scheduled at 6 weeks of age.

The neonatal nursery offers a very basic care level with possibility of phototherapy and intravenous infusion. Intubation and oxygen therapy was not possible at the time the trial was conducted. Admitted children did often share the available incubators. The service of the neonatal nursery is free, and children of all gestational ages are admitted. There is no possibility of transmission to a higher specialised unit.

### Enrolment

The study was initiated 20 February 2008. LBW children identified at the hospital were examined by a doctor or a trained nurse who also assessed maturity using Ballard score [16]. Anthropometric measurements were obtained and the child was examined. Eligible were boys with a weight below 2.5 kg. Exclusion criteria were major malformations, female sex, and weight at enrolment of ≥ 2500 g. Children who had already received BCG and children with clinical signs of vitamin A deficiency were also excluded, as were children that were too sick to be discharged by local standards. These children were referred for treatment*.* There was no age criterion, as all children weighing less than 2500 g and coming for their first vaccines were eligible. The oldest child enrolled was 64 days old, and the age distribution is described in Table 1. The mothers were informed of the study in the local language, Creole, and got a written explanation of the study in the official language, Portuguese. Oral and written consent was obtained. The mother signed the enrolment form if she could write, if not she put a fingerprint, and an independent observer signed the form. Provided consent, the mother drew a lot from a bag. The lot decided which treatment, VAS or OPV, her son would receive at enrolment. Randomisation was done in blocks of 24. The bags were prepared by the study supervisor; each bag contained 24 stapled lots in separate opaque envelopes. Twins were allocated the same treatment to prevent potential confusion regarding who had been vaccinated and supplemented. All mothers were encouraged to take their child to a health centre at 6 weeks of age to get BCG, OPV, and DTP. At every home visit the assistants checked the children’s vaccination cards and pointed out missing vaccines for the mothers to ensure that all children got OPV. Enrolment staff did not take part in the follow-up of the children.

**Table 1:** Baseline characteristics of the two randomisation groups

	**VAS at birth**	**OPV at birth**
	**(N = 116)**	**(N = 116)**
Enrolled in rainy season, n (%)	70 (60)	71 (61)
Enrolled at NH, n (%)	102 (88)	99 (85)
Living inside study area, n (%)	34 (29)	36 (31)
Twin, n (%)	26 (22)	24 (21)
Admission to neonatal nursery, n (%)	29 (25)	30 (26)
Age at inclusion, days (10–90 centiles)	2.5 (1–10)	2 (1–10)
Birth weight, kg (10–90 centiles)	2.21 (1.66-2.45)	2.22 (1.66-2.46)
Ballard score* (10–90 centiles)	36 (27–43)	36 (27–43)
Median maternal age, years (10–90 centiles)	23 (16–29)	22 (17–32)
Maternal schooling, n (%)		
None	40 (34)	38 (33)
<6 years	19 (16)	26 (22)
≥6 years	57 (49)	52 (45)
Electricity available, n (%)		
Yes	27 (23)	35 (30)
No	88 (76)	80 (69)
Unknown	1 (1)	1 (1)
Parity, n (%)		
1	62 (53)	57 (49)
2-3	30 (26)	34 (29)
>3	23 (20)	24 (21)
Unknown	1 (1)	1 (1)
Maternal MUAC, mm (10–90 centiles)	232 (208–276)	238 (208–284)

*Abbreviations:* NH National hospital; MUAC Mid upper arm circumference.

*Only available for children enrolled at the national hospital.

### Interventions

Vitamin A was given as a 0.5 ml oral supplement which was slowly released into the mouth of the child with a sterile syringe by a nurse. The supplement came in dark glass bottles that were prepared at Skanderborg Pharmacy, Denmark, and contained 20 doses of 25000 IU vitamin A as retinyl palmitate and 10 IU vitamin E per 0.5 ml oil. The bottles were kept at 2-8°C. Trivalent OPV was supplied through the national immunisation programme and administered orally. There was no blinding.

### Outcomes

#### ***Primary outcome: infant mortality***

**Figure 1:**
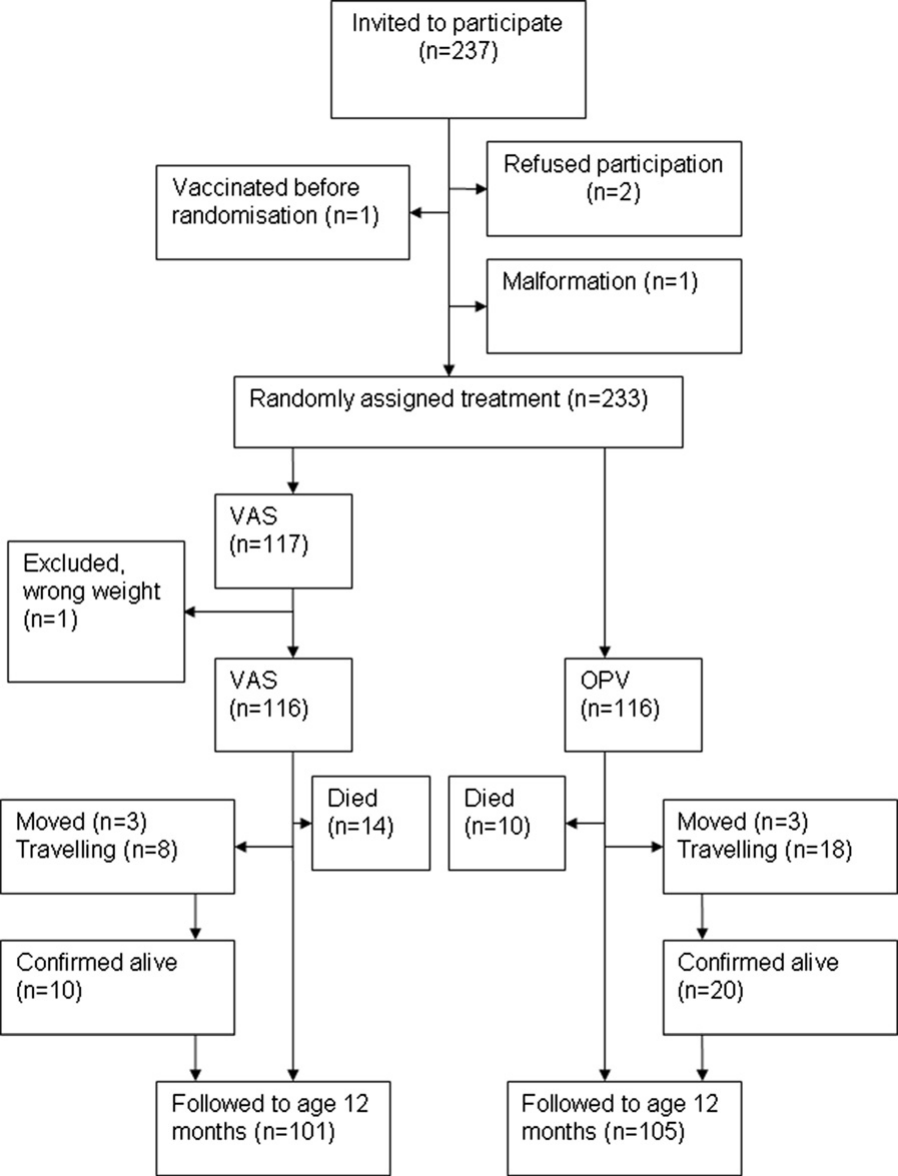
Trial profile.

The LBW children were visited within the first 3 days after enrolment, and children living inside the study area were visited on day 1–3 after enrolment to check for adverse events. All children who had not died, moved or were travelling were visited at 2, 6, and 12 months of age (Figure 1). The children living inside the BHP study area were furthermore followed by the HDSS. If the child moved outside Bissau or was absent at the visit, relatives or neighbours were asked if the child was still alive and how soon they would be told if the child died. Children travelling at 12 months were visited again at 15–18 months of age. When a death was registered, the assistant asked for the child’s health card. A verbal autopsy was conducted around three months after the death by a trained assistant. A local doctor read the autopsy and proposed a diagnosis. The cause of death in broad categories was determined later after reading the verbal autopsy and taking into account the local doctor’s diagnosis and possible hospital records.

We collected information on temperature, respiratory frequency, weight gain and a few other variables in the first three days after enrolment to be able to detect possible adverse effects of the intervention (which we did not find); however, we did not collect information on possible specific diagnoses of the surviving children enrolled in the trial.

#### ***Secondary outcome: growth***

A subgroup of children was visited biweekly for the first 3 months and at 4, 5, and 6 months of age by an anthropometry team measuring weight, length, and arm and head circumference. This sub study was initiated 10 April 2008 and continued enrolling children until the main trial was stopped at 18 November 2008. Measurements were made by two trained field assistants who visited the home of the child. The length of the child was measured supine using a measuring board (Seca Model 416). The weight of the undressed child was measured to the nearest 20 g using an electronic scale (Seca Model 835/336). Middle upper arm circumference (MUAC) and head circumference were measured using a TALC insertion tape. Children who were temporarily absent were visited later the same or the following day, whereas children travelling were only visited at the following round. Children who moved were localised as described above.

### Sample size considerations

We expected to enrol 900 boys in three years. With a mortality of 15% between enrolment and 12 months of age, we had 80% power to detect a 40% reduction in mortality for boys with a confidence level of 95%. With a sample size of 300 boys in the growth study, we should be able to detect a weight difference of 150 g in favor of the proposed versus the current policy with a power of 80% and a one-sided alpha of 0.05.

### Special investigations initiated after the identification of the cluster

Due to the cluster of deaths described in this paper, one of the authors (NL) supervised the verbal autopsies of the children. In November and December 2008, after the cluster was identified, we took 20 throat swabs from children currently treated at the neonatal nursery to search for viruses. The sample was collected with a cotton swab from the back of the child’s throat and placed in an Eppendorff tube containing 1 mL of alcohol. The tubes were stored at room temperature until analysis at Statens Serum Institut, Denmark. The samples were examined for Influenza A and B, Respiratory Syncytial Virus, Human Metapneumovirus, Parainfluenza, Adeno, Corona, Rhino, Entero, and Parecho viruses using PCR on a MagNaPure system. However, when the samples were collected there was no longer a mortality problem at the nursery and nothing was found in the throat swabs. Likewise we conducted immunological examinations of cytokine responses among children recruited in October and November. Few children were included and the cluster of deaths had passed. Hence, the results were unrevealing.

### Statistical methods

Statistical analysis was performed using Stata 11.2 software (Stata Corporation, College Station, TX). Baseline characteristics of children in the VAS group vs. children in the OPV group were compared using logistic or linear regression.

We used Cox regression to calculate Hazard Ratios (HR) for mortality with 95% Confidence Intervals (CI). Robust standard errors were used to account for interdependency of outcome between twins. Age was used as the underlying time and was thus inherently controlled for in the mortality analyses. Test for proportionality of hazard rates were computed using Schoenfeldt residuals and by visual inspection of the cumulative risk curves. Cumulative mortality curves were drawn using the Kaplan-Meier method. We tested whether there were differences in the age at death in a linear regression model on the log-transformed age.

We tested interactions between baseline characteristics, season of enrolment (rainy season June to November, dry season December to May), and admission to neonatal nursery before enrolment by Wald test statistics. We analysed effect modification by investigating the homogeneity of the effect of the intervention in the different categories of the suspected modifier, also by Wald test statistics. Effect modifiers considered were age at and place of enrolment, place of residence, birth weight, head circumference, MUAC, and maternal MUAC, age, parity, schooling, and socioeconomic status.

We compared the individual anthropometry measurements to the 2006 WHO growth reference [17]. Z-scores for length-for-age, weight-for-age, head circumference for age, and mid-upper-arm-circumference (MUAC)-for-age (only available for children aged 12 weeks or more) were derived. Children were classified as stunted (length-for-age z-score *≤ -*2) and underweight (weight-for-age z-score *≤ -*2) at all time points. We compared differences in z-scores by linear regression. For variables that were not normally distributed, geometric mean ratios (GMRs) were calculated from the log-transformed variable. Differences in growth between baseline and 4 weeks visits were compared using linear regression. We calculated relative risks (RR) of being stunted or underweight in Poisson regression models with robust standard errors [18]. Possible interaction with season of inclusion was explored.

### Ethics statement

There have been no cases of poliomyelitis in Guinea-Bissau for at least a decade. As a “natural experiment” had worryingly shown that boys who had not received OPV at birth had significantly lower mortality than boys who had received OPV at birth [14], and as OPV is also provided at 6, 10 and 14 weeks of age and during national immunisation days, we found it ethically justified to conduct a trial not giving boys OPV at birth if they had been randomised to vitamin A. The protocol was approved by the Guinean Ministry of Health’s Research Coordination Committee, and the Danish Central Ethics Committee gave its consultative approval. The trial was registered at http://www.clinicaltrials.gov, identifier NCT00625482.

## Results

From 20 February 2008 to the trial was halted on 18 November 2008 a total of 237 boys were invited to participate. Two mothers refused participation, one child received vaccines before randomisation, and one child turned out not to be eligible due to a malformation. One child in the VAS group had weighed 2300 g at birth but had gained weight and weighed 2500 g at inclusion and was excluded from analysis (Figure 1). Hence, we ended up with 232 boys; 116 in the OPV group and 116 in the VAS group. As shown in Figure 1, at the 12 months visits, three children in each group had moved; however, only two of the children, one in each group, could not be confirmed alive. All travelling children were confirmed alive by relatives or neighbours.

Baseline characteristics of the two intervention groups are shown in Table 1. The medical examination made before enrolment showed no difference in heart frequency, respiratory frequency, or temperature between the cluster children and the non-cluster children enrolled from the neonatal nursery or from the maternity ward. Breastfeeding was initiated in all children. At the 2 months visit, all visited children were breastfed. At the 6 months visit, 4 children in the OPV group and 2 in the VAS group were not breastfed any more. Of these, one child (OPV) died before 12 months of age. At the 12 months visit, another 4 children had been weaned (1 OPV, 3 VAS).

### Mortality cluster

**Figure 2:**
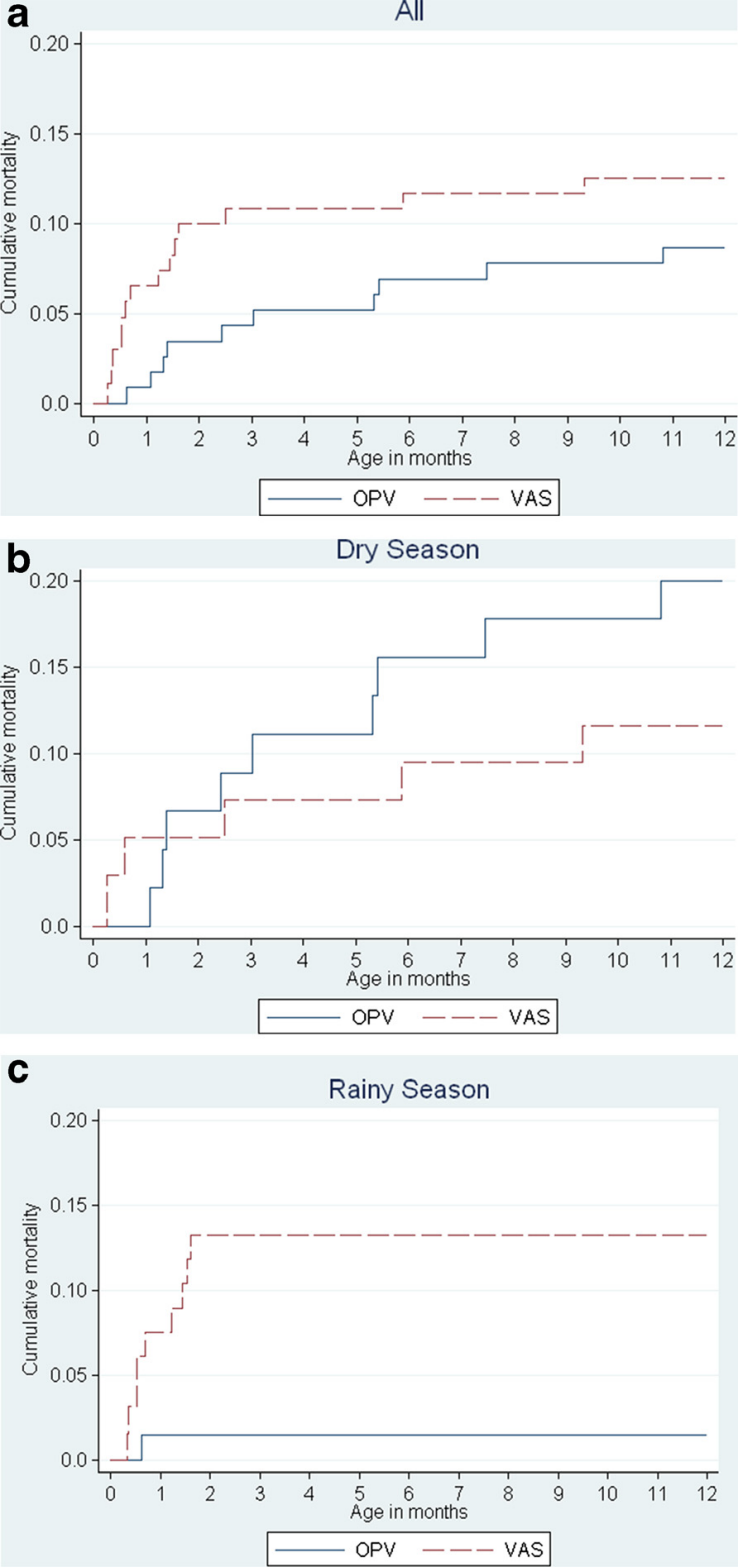
**Cumulative mortality curves as a function of receiving VAS or OPV. (a)** Overall, **(b)** Dry season, **(c)** Rainy season.

When several death forms were brought back by the anthropometric team and the team conducting the 2-months visits in October-November 2008, we compiled the mortality statistics shown in Table 2. Season was monitored because previous analyses had shown that though the overall effect of VAS appeared to be beneficial for boys, there might not be a beneficial effect in the rainy season. This was strongly supported by the incoming reports; there was 10-fold increased mortality among boys receiving VAS in the rainy season and a clear inversion of the pattern between dry and rainy season (Table 2). Based on these data we decided to temporarily halt the enrolment of LBW boys on 18 November 2008. As shown in Figure 2a, mortality in the group receiving VAS was 7 fold higher in the first month of life (HR = 7.20 (95% Confidence Interval (CI): 0.89 – 58.5)) and 3 fold higher at 2 months of age (HR = 2.85 (0.91 – 8.93)).

**Table 2:** The effect of VAS/OPV at birth on infant mortality overall and by season of enrolment

	**Censored at 18 November 2008**			**With full follow up time**		
	**VAS**	**OPV**	**VAS vs. OPV**	**VAS**	**OPV**	**VAS vs. OPV**
	**MR**	**MR**	**HR (95% CI)**	**MR**	**MR**	**HR (95% CI)**
	**(Deaths/pyrs)**	**(Deaths/pyrs)**		**(Deaths/pyrs)**	**(Deaths/pyrs)**	
All	35.1	21.2	1.66 (0.68 – 4.02)	13.6	9.3	1.46 (0.65 - 3.29)
	13/37	8/37		(14/103)	(10/108)	
By season						
Dry	15.8	28.9	0.54 (0.16 - 1.84)	11.9	23.3	0.53 (0.18 - 1.55)
	4/25	7/24		(5/42)	(9/39)	
Rainy	77.1	7.4	10.0 (1.24 – 80.5)	14.9	1.4	9.91 (1.23 - 79.8)
	9/12	1/14		(9/61)	(1/69)	
P for interaction			0.02			0.02

MR per 100 years of follow up.

*Abbreviations:* Pyrs person years of follow up.

Subsequent examinations showed that the children who died had not received the same bottle of VAS and common contamination was therefore unlikely. However, the examination revealed that most of the children who died had been in the neonatal nursery (Table 3). Of the 14 boys admitted to the neonatal nursery during September 2008, two died before being discharged; of the 12 boys being discharged and enrolled in the present trial, six received VAS of whom five died whereas six received OPV of whom one died within two months of age (p = 0.05). One VAS boy enrolled in September who also died within the first 2 months of life had not himself been admitted to the neonatal nursery, but his twin had. These seven boys were between 9 and 43 days old when they died; the median age was 18 days. Among the additional children enrolled at all enrolment sites in October and November before we halted the study, there were no deaths (Table 3).

**Table 3:** The fraction of dead/enrolled children by place of enrolment, month of enrolment, and randomisation group

	**No. of deaths/enrolled (% dead)**			
	**Enrolled at neonatal nursery**		**Enrolled at maternity ward or health centres**	
**Month of enrolment**	**VAS**	**OPV**	**VAS**	**OPV**
February	0/0 (0)	1/2 (50)	0/4 (0)	1/4 (25)
March	2/3 (67)	0/4 (0)	0/11 (0)	1/10 (10)
April	2/6 (33)	1/4 (25)	0/8 (0)	1/10 (10)
May	0/5 (0)	3/4 (75)	1/9 (11)	1/7 (14)
Total, dry season	4/14 (29)	5/14 (36)	1/32 (3)	4/31 (13)
June	0/2 (0)	0/4 (0)	0/5 (0)	0/6 (0)
July	1/3 (33)	0/1 (0)	1/6 (17)	0/8 (0)
August	1/2 (50)	0/2 (0)	0/8 (0)	0/5 (0)
September	5/6 (83)	1/6 (17)	1/9 (11)	0/9 (0)
October	0/4 (0)	0/0 (0)	0/17 (0)	0/20 (0)
November	0/0 (0)	0/3 (0)	0/8 (0)	0/7 (0)
Total, rainy season	7/17 (41)	1/16 (6)	2/53 (4)	0/55 (0)
Total	11/31 (35)	6/30 (20)	3/85 (4)	4/86 (5)

### Main outcome: infant mortality

Followed to 12 months of age the mortality rate was 11.4 deaths per 100 person-years, somewhat lower than the anticipated 15/100 (Figure 2a-c). Fourteen VAS boys and 10 OPV boys died resulting in a HR of 1.46 (0.65 – 3.29) (Table 2). The estimates did not change if children who moved or were travelling were censored at the day they left. At 2 and 6 months of age the HR for VAS vs. OPV were 2.85 (0.91 – 8.93) and 1.69 (0.70 – 4.09), respectively.

All deaths among children included in the rainy season occurred before two months of age and VAS boys were therefore overall younger than OPV boys when they died (median age at death of 28 days in VAS boys and 82 days in OPV boys, p = 0.04).

The number of very low birth weight babies (VLBW, birth weight < 1500 g) was 7 (6%) in each group. Two of the VLBW babies in the OPV group died during follow up, one of them was enrolled at the neonatal nursery during September 2008 (the cluster period). Another VLBW baby in the OPV group was also enrolled from the neonatal nursery during this period but survived. Three VLBW babies in the VAS group died during follow up, one of them was enrolled from the neonatal nursery in the cluster period. None of the other VLBW babies in the VAS group were enrolled during the cluster period.

### Causes of death

Of the seven dead children enrolled in the cluster period, four (all VAS) died from respiratory diseases (Table 4). One child (OPV) died from kernicterus, and the cause of death could not be established in two children (VAS).

**Table 4:** Causes of death by intervention and age at death

	**Deaths within first 2 months**		**Deaths after 2 months of age**	
	**VAS**	**OPV**	**VAS**	**OPV**
Resp. diseases	5	1	2	1
Diarrhoea	2	0	0	0
Sepsis	1	0	0	1
Kernicterus	0	1	0	0
Chickenpox	0	0	0	1
Unknown*	3	2	1	3
Total	11	4	3	6

*Three families moved before autopsy, and a diagnosis could not be established in six children*.*

### Secondary outcomes: growth

Eighty-six children from the OPV group and 87 from the VAS group were enrolled in the anthropometry sub study; 82 and 77 children, respectively, had at least one visit. An average of 74% of the children was found at home at each visit. Of the 173 children enrolled in the anthropometry study, nine VAS and two OPV boys died before the last anthropometry visit at 6 months after enrolment, corresponding to a relative risk (RR) of loss to follow up due to death for VAS vs. OPV of 4.44 (0.99 – 20.08).

In the subgroup followed for growth, more children in the VAS group were stunted at baseline. We therefore adjusted length measures at the following visits for being stunted at baseline in analyses where adjustment changed the estimate by more than 10%. Two weeks after enrolment VAS children were significantly lighter and had a lower weight-for-age z-score and MUAC than OPV children. These differences were also found at the 4, 6, 10, and 12 week visits (data only shown for the 4 weeks visit, Table 5). There were no differences in length and head circumference between the two groups at any visit when length analyses were controlled for being stunted at baseline. At 6 months VAS children were more often underweight than OPV children (Table 5). Because of the imbalance of stunted children between the two groups, we studied growth between baseline and the 4 weeks visit. It turned out that even though stunted children, regardless of randomisation group, experienced significantly better linear growth than non-stunted children between baseline and 4 weeks, probably reflecting a catch up growth, VAS children had a significantly poorer linear growth (Difference adjusted for being stunted at baseline = -1.02 (-1.66; -0.38)) between baseline and 4 weeks). There was no interaction between growth and season (data not shown).

**Table 5:** The effect of VAS/OPV on anthropometric parameters at baseline, 4 weeks, and 6 months after enrolment

	**Baseline**				**4 weeks**				**6 months**			
	**VAS**	**OPV**	**Difference**^**#**^**/GMR**^**¤**^	**RR**	**VAS**	**OPV**	**Difference**^**#**^**/GMR**^**¤**^	**RR**	**VAS**	**OPV**	**Difference**	**RR**
	**N = 77**	**N = 82**	**(95% CI)**	**(95% CI)**	**N = 63**	**N = 66**	**(95% CI)**	**(95% CI)**	**N = 67**	**N = 73**	**(95% CI)**	**(95% CI)**
Age, days	2	2			32	34			183	183		
Length,	44.9	45.2	0.99^¤^		48.3	49.5	0.45^¤^		63.0	63.6	-0.32	
cm*			(0.98-1.01)				(0.17-1.16)				(-1.31;0.67)	
LAZ*	-3.01	-2.84	-0.17^#^		-3.38	-2.89	-0.28^#^		-2.16	-1.93	-0.13	
			(-0.54;0.20)				(-0.77;0.22)				(-0.59;0.34)	
Stunted*	85%	72%		**1.19**	86%	74%		1.12	55%	37%		1.49
				**(1.00-1.40)**				(0.94-1.33)				(0.98-2.23)
Weight,	2.05	2.13	0.96^¤^		2.84	3.10	-**0.26**^**#**^		6.51	6.57	-0.06	
kg			(0.92-1.01)				**(-0.46;-0.07)**				(-0.43;0.31)	
WAZ	-2.95	-2.73	-0.20^#^		-3.31	-3.00	**-0.46**^**#**^		-3.20	-3.06	-0.11	
			(-0.47;0.08)				**(-0.88;-0.04)**				(-0.61;0.39)	
Under-	97%	99%		0.99	87%	73%		**1.20**	48%	29%		**1.68**
weight				(0.94-1.03)				**(1.01-1.43)**				**(1.04-2.73)**
HC, cm	31.0	31.3	-0.27^#^		35.3	35.6	-0.22^#^		42.6	42.8	-0.16	
			(-0.85;0.31)				(-0.76;0.32)				(-0.72;0.40)	
HCAZ	-3.11	-2.89	-0.22^#^		-1.86	-1.47	-0.09^#^		-0.55	-0.43	-0.13	
			(-0.71;0.26)				(-0.55;0.37)				(-0.58;0.33)	
MUAC,	7.7	7.9	0.80^¤^		9.4	10.1	**-0.50**^**#**^		13.9	14.1	-0.21	
cm			(0.63-1.02)				**(-0.93;-0.07)**				(-0.65;0.23)	
ACAZ	-	-			-	-	-		-0.35	-0.15	-0.19	
											(-0.62;0.23)	

*Length measures are adjusted for being stunted at baseline.

^¤^Geometric mean ratio (GMR) is provided for non-normally distributed data, ^#^Difference for normally distributed data.

*Abbreviations:* RR relative risk; LAZ length-for-age z-score; WAZ weight-for-age z-score; HC head circumference; HCAZ head circumference-for-age z-score; MUAC middle upper arm circumference; ACAZ Arm circumference-for-age z-score.

Significant values in **bold***.*

## Discussion

### Principal findings

A cluster of deaths occurred during the rainy season among the boys enrolled in the trial of VAS versus OPV and affected primarily those who had received VAS. The effect of VAS versus OPV differed significantly between the dry and the rainy season with a 10-fold higher mortality in the rainy season. VAS recipients had a significantly poorer growth measured by weight and MUAC up to 3 months after enrolment.

### Strengths and weaknesses

The close follow up of LBW children has been conducted since 2002 by the same staff. The trial had to be stopped prematurely due to the cluster of deaths and the study therefore did not reach the anticipated sample size.

### Mortality

A sudden increase in deaths among boys who had received VAS in the rainy season provoked our attention and the decision to halt inclusion. We subsequently detected that these boys had all been at the neonatal nursery within the same week. The deaths were mainly due to respiratory problems. Overall the study sample size was clearly too small to make firm conclusions on the effect of receiving VAS versus placebo, but it is noteworthy that there were a quite strong interaction between VAS and season, with a tendency for a beneficial effect in the dry season, but a significant negative effect in the rainy season.

### Growth

We found worse growth for the VAS recipients than the OPV recipients in the first months of life irrespective of season. We have studied the effect of neonatal VAS given with BCG at birth and found a beneficial effect on growth for boys [19]. Also, a trial from Indonesia showed a beneficial overall effect of neonatal VAS on growth up to 3 years of age [20]. Another trial from Java, Indonesia, found complex interactions between VAS and season in children aged 6–48 months at supplementation with the least beneficial effect of VAS in seasons with a high burden of respiratory diseases [21]. However, VAS was not harmful. The effect of OPV0 on growth has not been studied before.

### Chance or cluster

The sudden increase in the number of deaths among boys who had received VAS and who had been in contact with the neonatal nursery made us speculate that they had been infected with a pathogen that either interacted negatively with VAS or was dealt better with by OPV vaccinated boys. A pathogen could easily have spread among the children through the suboptimal hygienic conditions. We could not identify any likely pathogen or immunological differences between the two groups which could explain the cluster, but this is perhaps not surprising as the mortality was no longer elevated at the time when we collected throat swaps and immunological samples. The pathological pictures of the dead children were quite different and the deaths did not occur immediately. Hence, it is unlikely that the children died of the same infection. However, it may be speculated that the pathogen weakened the children who died later, possibly by encounter with a new pathogen.

When we halted the trial we did not know whether there might be more deaths among children with whom we had not yet had contact. However, that was not the case; there were no additional early deaths among the children recruited in October and November. The problem apparently had passed. However, we did not restart the trial. Though this may have been due to a pathogen which was no longer present there was no reason to risk that the same might happen again. Furthermore, the boys who had received OPV0 clearly grew better in the first months of life than the boys who had received neonatal VAS. Hence, there was no indication that it was relevant to continue the trial.

With the study design it cannot be determined whether vitamin A was harmful or whether OPV stimulated a non-specific immune response which provided some protection against infections as also seen for other live vaccines [22].

When we initiated the trial, all available data suggested that neonatal VAS be beneficial for boys. However, subsequently data from Zimbabwe have been published, showing a 19% increase in mortality for boys [23]. Though the results are not directly comparable with the other trials because the trial was 2-by-2 factorial with provision of maternal VAS as well, and because the prevalence of HIV was very high and most deaths occurred in children of HIV positive mothers, the results nonetheless show that neonatal VAS can be harmful to boys under certain circumstances. We have previously found strong seasonal differences in the response to neonatal VAS [1]. In boys VAS had a strong beneficial effect in the dry season (0.45 (0.24 – 0.84)) but tended to have a negative effect in the rainy season 1.53 (0.84 – 2.79). This could be seen as support of a negative effect of VAS also in the present study, though it should be noted that no negative effect was seen in our other studies [2,24]. Hence we cannot rule out that neonatal VAS had a negative effect for boys in the present trial.

However, we are more inclined to believe that OPV0 was particularly beneficial. Though we have previously found increased male mortality after OPV0, it was based on an observational study and it is contradicted by other studies on OPV [25,26]. Observations from Chile and Brazil showed significant reduction in infantile diarrhoea mortality following the first massive vaccination campaigns with OPV [27,28]. A recent study from Finland found that children who had received OPV had fewer episodes of otitis media at age 6–18 months than control children who received inactivated polio vaccine (IPV) [29].

The growth data support a beneficial effect of OPV0 rather than a negative effect of VAS since the differences in growth between the two groups gradually disappeared as more children in both groups got OPV1 scheduled to be given at 6 weeks of age.

Hence, rather than neonatal VAS being bad, we are more in favour of the hypothesis that the immunostimulation provided by OPV may have protected the children in the OPV group against pathogens circulating possibly by priming a Th1 type immune response, as hypothesised in several studies [30-32].

## Conclusion

These observations may be important. The introduction of neonatal VAS is debated [33-39] and WHO has launched three mega trials of neonatal VAS with more than 100,000 children to inform global policy. The present study does not support a policy of providing VAS, but clearly it cannot be seen as strong evidence against this policy on its own rights.

Though OPV0 is official policy, many African children do not receive it [40]; for example, there are often special rules not to give OPV0 after two weeks of age. Furthermore, there are long-term plans to replace OPV with inactivated polio vaccine (IPV) since OPV is associated with a small risk of developing polio paralysis [41]. If OPV has beneficial non-specific effects as suggested by this and other studies [25,26,29,42], replacing OPV with IPV may not have a beneficial effect on overall survival. For example, we found that among children randomised to IPV as a control vaccine, girls had significantly higher mortality than the boys [43].

In conclusion, receiving VAS at birth instead of OPV was not beneficial for the LBW boys in this trial. Growth in the first few months of life was affected negatively and there was a tendency for higher mortality during the first weeks of life which was statistically significant in the rainy season. With the premature closure of the trial, however, the trial was clearly underpowered to establish a causal relation between the intervention and the outcomes, and the results cannot be generalised. We think it is most likely that OPV at birth provided a non-specific immune stimulation that proved beneficial in dealing with a circulating respiratory pathogen in the rainy season. However, the results of this study call for extra caution when testing the effect of NVAS in the future.

## Abbreviations

BCG: Bacille calmette-guerin; BHP: Bandim health project; CI: Confidence interval; DTP: Diphtheria-tetanus-pertussis vaccine; GMR: Geometric mean ratio; GPS: Global positioning system; HDSS: Health and demographic surveillance system; HR: Hazard ratio; IPV: Inactivated polio vaccine; IU: International units; LBW: Low-birth weight; MUAC: Mid-upper-arm-circumference; NVAS: Neonatal vitamin A supplementation; OPV0: OPV at birth; PCR: Polymerase chain reaction; PI: Primary investigator; RR: Relative risk; VAS: Vitamin A supplementation; WHO: World Health Organization.

## Competing interests

The authors declare that they have no competing interests.

## Authors’ contributions

CSB and PA designed the study. SB-S, CSB, and PA initiated the study. SB-S, NL, MJJ, LC and IM were responsible for the recruitment and follow-up of participants. NL and AA were responsible for the statistical analysis, and NL wrote the first draft of the paper. All authors contributed to and approved the final version of the paper.

## Pre-publication history

The pre-publication history for this paper can be accessed here:

http://www.biomedcentral.com/1471-2431/14/214/prepub
